# The Core Gut Microbiome of Black Soldier Fly (*Hermetia illucens*) Larvae Raised on Low-Bioburden Diets

**DOI:** 10.3389/fmicb.2020.00993

**Published:** 2020-05-21

**Authors:** Thomas Klammsteiner, Andreas Walter, Tajda Bogataj, Carina D. Heussler, Blaž Stres, Florian M. Steiner, Birgit C. Schlick-Steiner, Wolfgang Arthofer, Heribert Insam

**Affiliations:** ^1^Department of Microbiology, Faculty of Biology, University of Innsbruck, Innsbruck, Austria; ^2^Department of Environmental, Process and Energy Engineering, MCI – The Entrepreneurial School, Innsbruck, Austria; ^3^Department of Biotechnology and Food Engineering, MCI – The Entrepreneurial School, Innsbruck, Austria; ^4^Department of Ecology, Faculty of Biology, University of Innsbruck, Innsbruck, Austria; ^5^Department of Animal Science, Biotechnical Faculty, University of Ljubljana, Ljubljana, Slovenia; ^6^Institute of Sanitary Engineering, Faculty of Geodetic and Civil Engineering, University of Ljubljana, Ljubljana, Slovenia; ^7^Faculty of Medicine, University of Ljubljana, Ljubljana, Slovenia

**Keywords:** *Actinomyces*, animal feedstuff, waste valorization, circular economy, microbial communities, larval metabolism, 16S amplicon sequencing

## Abstract

An organism’s gut microbiome handles most of the metabolic processes associated with food intake and digestion but can also strongly affect health and behavior. A stable microbial core community in the gut provides general metabolic competences for substrate degradation and is robust against extrinsic disturbances like changing diets or pathogens. Black Soldier Fly larvae (BSFL; *Hermetia illucens*) are well known for their ability to efficiently degrade a wide spectrum of organic materials. The ingested substrates build up the high fat and protein content in their bodies that make the larvae interesting for the animal feedstuff industry. In this study, we subjected BSFL to three distinct types of diets carrying a low bioburden and assessed the diets’ impact on larval development and on the composition of the bacterial and archaeal gut community. No significant impact on the gut microbiome across treatments pointed us to the presence of a predominant core community backed by a diverse spectrum of low-abundance taxa. *Actinomyces* spp., *Dysgonomonas* spp., and *Enterococcus* spp. as main members of this community provide various functional and metabolic skills that could be crucial for the thriving of BSFL in various environments. This indicates that the type of diet could play a lesser role in guts of BSFL than previously assumed and that instead a stable autochthonous collection of bacteria provides the tools for degrading of a broad range of substrates. Characterizing the interplay between the core gut microbiome and BSFL helps to understand the involved degradation processes and could contribute to further improving large-scale BSFL rearing.

## Introduction

Diet is known to shape structure and function of an organism’s gut microbiota (David et al., 2014). In many cases, a major part of metazoan digestive capabilities root in its gut-residing aggregation of a mostly prokaryotic microbiota (Rajagopal, 2009; Ursell et al., 2012; Engel and Moran, 2013). Within a taxonomic entity, very often a microbial core community may be identified, ensuring essential catabolic aptitudes for health and survival (Raymann and Moran, 2018). Maintaining the stability of such a community may provide protection against extrinsic microbial advances, facilitate degradation, and support the thriving of an organism (Dillon and Dillon, 2004; Engel and Moran, 2013).

The Black Soldier Fly (*Hermetia illucens*; BSF) is known as a workhorse when it comes to valorizing biodegradable organic wastes (Clariza Samayoa et al., 2016). Rapid growth, broad degradation capabilities, and non-competence as a vector for human diseases make the fly ideal for industrial applications (Tomberlin et al., 2002; Wang and Shelomi, 2017). Larvae transitioning to the prepupal stage move out of the humid organic waste and seek a dry spot for pupation. This “self-harvesting” property facilitates the automatization of larva collection (Sheppard et al., 2002). The larvae shine with a high fat (>35%) and protein (>40%) content featuring a favorable amino acid spectrum for application as animal feed (Cullere et al., 2016; Surendra et al., 2016; Ushakova et al., 2016). Thus, larvae and products thereof increasingly find use in pisciculture and poultry farming (Wang and Shelomi, 2017), and more concrete EU-wide regulations regarding the use of insects and derivatives as animal feed are in the works (European Parliament and of the Council, 2015).

The larvae’s ability to produce antimicrobial peptides repressing the number of pathogenic and supposedly other bacteria in their environment is still an object of study but could act as a barrier for extrinsic microbial colonization of the larvae and supports the maintenance of an inborn microbial gut community (Erickson et al., 2004; Choi et al., 2012; Park et al., 2015). The growing insect industry and increasing use of insect products demand an in-depth understanding not only of the fly’s biology but also of the involved microbial networks to standardize rearing methods, improve waste degradation, maximize biomass output, and meet the hygienic standards (Pastor et al., 2015; Boccazzi et al., 2017; De Smet et al., 2018). Recently, Wynants et al. (2018) observed strong variations in the composition of microbial gut communities in larvae across different rearing locations. Although many biotic and abiotic factors influence the specific microbiome structure, these authors were able to detect bacteria like *Morganella* sp., *Enterococcus* sp., *Pseudomonas* spp., *Providencia* sp., and Bacillaceae in multiple independent samples.

In this study, we investigated the impact of three diets [chickenfeed (CF), grass-cuttings (GC), fruit/vegetables (FV)] carrying a low bioburden, compared with waste-related substrates, on the microbial community composition during larval development. Besides CF as a control diet, we selected these diets because produce and green waste account for a large fraction of common municipal household wastes (European Environment Agency, 2013) and should be considered for BSF treatment. We hypothesized that larvae would perform differently with respect to biomass gain and developmental progress and presumed that a diet-independent association of microbial gut colonizers could support versatile larval growth. Our analysis focused on the characterization of a core microbial community uninfluenced by time or diet, which acts as a foundation for degradative activities in the larval gut.

## Materials and Methods

### Rearing of Larvae and Experimental Set-Up

Larvae of *H. illucens* were obtained from Illucens (Ahaus, Germany) in 2014 and were kept as laboratory population under stable environmental conditions in a Fitotron^®^ SGC 120 (Weiss Technik, United Kingdom) climate chamber [27°C and 60% relative humidity (RH), 16 h photoperiod with light settings as described in Heussler et al., 2018] in Innsbruck, Austria. The population was maintained on a diet based on a 2:3 mixture of ground CF (Grünes Legekorn Premium, Unser Lagerhaus WHG, Austria) processed with a Fidibus flour mill, Komo Mills, Austria) and water. Larvae were kept in 30 × 15 × 5 cm non-transparent plastic boxes, covered with nets for aeration and containing sterilized pine humus (PH; *ad libitum*) as litter for humidity regulation. Until eclosion, pupae were stored in non-transparent plastic cups covered with nets containing straw litter. Flies were kept for oviposition in 50 × 30 × 25 cm transparent plastic boxes with corrugated plastic strips. Two-hundred 6-day old larvae were used for each replicate (three replicates per treatment). After hatching, the larvae were fed with CF for 6 days before being introduced to the two new diets (GC and FV mix). Feeding with CF continued in a control group consisting of three replicates.

### Substrate Preparation and Feed Amounts

Chickenfeed was used as control diet, prepared as a 2:3 mixture of ground CF and water as for the standard population maintenance. For GC, freshly cut grass from a lawn mower collector box was further shredded using a Vitamix TNC^®^ electric blender (Vitamix, Germany). The FV diet consisted of cucumber, tomato, orange, and apple, prepared in a ratio of 0.5:1:1:1 (fw/fw), and the ingredients were manually minced. Prior to use, the water content of the substrates was equalized based on their dry weight. Feed was added every 3 days and the feed amounts were set to 100, 300, and 400 mg larva^–1^ for CF, FV, and GC, respectively, based on the organic content of the substrate. The efficiency of substrate degradation and the conversion to larval biomass were calculated based on (1) and (2).

(1) WRI=Dt⁢x⁢ 100⁢D=W-RW

Waste reduction index (WRI): W = total amount of organic material, R = residue after time t, t = days larvae were fed with material (Diener et al., 2009).

(2) $$\mathrm{ECD}=\frac{\text{B}}{\left(I-F\right)}\,B=\left(I-F\right)-M$$

Efficiency of conversion of digested food (ECD): B = assimilated food used for growth (measured as prepupal biomass), I = total food offered, F = residues in boxes, M = metabolized food (based on mass balance) (Diener et al., 2009).

### Sampling

Twenty larvae were removed every 3 days from each box and constituted a sample. Larval fresh, dry, and organic dry matter were determined, and the gut was extracted (for details, see section “Gut Removal and DNA-Extraction”). Simultaneously, 1 g aliquots of homogenously mixed residual matter (containing substrate residues, pine humus, and excrements) were taken from each box for the determination of physicochemical parameters.

### Physicochemical Parameters

During the experiment, a temperature of 27°C and RH of 60% were maintained and constantly monitored. The residual-matter samples were suspended in 9 ml 0.0125 M CaCl_2_ for the determination of ammonium (NH_4_^+^) and in 9 ml distilled water for pH measurement. The suspensions were agitated at 150 r/min for 60 min (Controlled Environment Incubator Shaker, New Brunswick Scientific, United States) and subsequently filtered through a folded filter (MN 615 1/4 150 mm, Macherey-Nagel^TM^). The extracts were stored at 4°C overnight. Ammonium concentration was measured every 3 days until the termination of the experiment using the NANOCOLOR^®^ Ammonium 50 Kit (Macherey-Nagel, Germany). The pH was measured using a Metrohm 744 pH Meter (Metrohm Inula, Switzerland).

### Initial Substrate Characterization

Dry matter and water content were determined by calculating the difference between the fresh weight of samples and the sample weight after oven-drying at 105°C for 24 h. Organic dry matter was determined by combusting finely ground dried samples in a muffle furnace (Carbolite, CWF 1000) at 550°C for 5 h and determining the residual weight.

Aliquots of the oven-dried samples were used to determine total carbon and nitrogen with a Leco TruSpec CHN Elemental Determinator (Leco, United States) following the manufacturer’s protocol.

As preparation for the measurement of unbound fat, substrate samples were weighed into 15-ml plastic tubes (Sarstedt, Germany), mixed with 5 ml water, and centrifuged at 11,000 × *g* for 20 min. The liquid phase was recovered, oven-dried at 105°C for 24 h, and the residues were weighed. The fat content was calculated as follows (3):

(3) $$\mathrm{Fat}\left(\%\right)=\frac{\mathrm{liquid}\,\mathrm{phase}-\mathrm{sample}\,\mathrm{dry}\,\mathrm{weight}}{\mathrm{sample}\,\mathrm{fresh}\,\mathrm{weight}}*100$$

To determine chemical oxygen demand (COD) and ammonium and protein content, 10-g aliquots of substrate samples were mixed with 25 ml of deionized water and vortexed for a few seconds. After a 30-min incubation at room temperature, the samples were shaken at 25°C and 120 r/min (Controlled Environment Incubator Shaker, New Brunswick Scientific, United States) for another 30 min before subsequent filtration (MN 615 1/4 150 mm folded filters, Macherey-Nagel^TM^). The COD and ammonium concentration in the filtrates were measured using the NANOCOLOR^®^ COD 1500 kit and Ammonium 50 kit (Macherey-Nagel, Germany), respectively. Protein content was determined by the Lowry (alkaline copper reduction) assay according to Noble and Bailey (2009).

The DNA extraction of the filtered substrate samples was carried out using the NucleoSpin^®^ Soil-Kit (Macherey-Nagel, Germany) following the manufacturer’s protocol. The extracts contained 18.2 ± 10.3 ng μl^–1^ DNA and were stored at 4°C until sequencing.

### Gut Removal and DNA-Extraction

A total of 36 gut samples were collected during the feeding experiments. They were extracted in triplicates at five time points (Day_0,3,9,15,21_), whereas samples of Day_0_ derived from the collective initial population before the separation of larvae and the introduction of new diets. At Day_21_, only guts from GC and FV treatments were extracted as CF fed larvae already transitioned to prepupal stage at Day_15_. Prior to the removal of the gut, the larvae were kept at −20°C for 30 min for devitalization. After disinfecting the thawed larval surfaces with 70% ethanol, a few millimeters of the anterior part were cut off using a sterile scalpel. The guts were pulled out using sterile forceps, transferred into a sterile microcentrifuge tube (minimum 0.05 g gut tissue/replicate), and snap frozen until further use.

After thawing the gut samples, the DNA was extracted using the NucleoSpin^®^ Soil-Kit (Macherey-Nagel Germany) following the manufacturer’s protocol with some modifications: one additional glass (Ø = 1.0 mm) and three additional silicon beads (Ø = 0.5 mm) were added to each bead tube and subsequently autoclaved. To each tube, 0.05 g of gut sample, 700 μl Buffer SL1 and 150 μl Enhancer SX were added before incubating them at 97°C and 400 r/min for 20 min. After this homogenization-step, the tubes were vortexed at high speed for 5 min and centrifuged at 11,000 × *g* for 2 min. Next, 150 μl Buffer SL3 were added and the tubes were vortexed for 5 s. After another incubation-step at 4°C for 5 min, the samples were centrifuged for 1 min at 11,000 × *g*. The steps for binding and washing were executed as described in the manual. The gut samples contained 22.8 ± 9.6 ng μl^–1^ DNA and were stored at 4°C until sequencing.

### Next Generation Sequencing

Illumina MiSeq amplicon sequencing using the 2 × 250 base pairs paired-end approach was performed by Microsynth AG (Balgach, Switzerland) with universal bacterial/archaeal primers 515f (GTGCCAGCMGCCGCGGTAA) and 806r (GGACTACHVGGGTWTCTAAT) targeting the V4 region on the 16S rRNA of 36 gut samples (Caporaso et al., 2011). Samples of each time-point and treatment were analyzed in triplicates. Microsynth AG provided library preparation based on Nextera two-step PCR including purification and pooling, demultiplexing, removal of adaptors and primers, and stitching of trimmed reads.

### Data Processing

Trimmed raw reads obtained from Illumina MiSeq amplicon sequencing were analyzed using mothur 1.40.0. (Schloss et al., 2009), and sequences were aligned to a V4-trimmed version of the SILVA reference database v.132 (Quast et al., 2013). Following the MiSeq SOP (Kozich et al., 2013), ambiguous bases were removed and a maximum number of eight homopolymers was allowed to reduce sequencing errors. Chimeric sequences were removed using the chimera uchime algorithm implemented in mothur (Edgar et al., 2011). For the construction of the bacterial OTU dataset, all other lineages (eukaryotes, chloroplasts, mitochondria, archaea, unknown) were removed, and bacterial sequences were subsequently *de novo* clustered based on 97% similarity. For the taxonomic focus, closed-reference phylotype clustering on genus level was applied. For the archaeal dataset, all sequences assigned to archaea were filtered from the dataset and further *de novo* clustered on 97% similarity and genus level phylotypes. OTUs not present in at least five samples were removed from the datasets prior to statistical analysis.

All statistical tests were carried out at a significance level of α = 0.05. Analyses of molecular variance (AMOVA) and homogeneity of molecular variance (HOMOVA) were performed in mothur on grouped triplicates of each sampling time-point, separated by treatment. One- and two-way non-parametric multivariate analysis of variance (NPMANOVA) were calculated in PAST (v.2.17c; Hammer et al., 2001) to detect significant differences between sampling time-points and substrates and to investigate the general influence of substrate and time variables on the spread of data. Alpha diversity measurements based on Chao1 species richness estimator and Shannon diversity index were conducted in R v.3.6.0 (R Core Team, 2018) using the phyloseq (McMurdie and Holmes, 2013) and vegan (Oksanen et al., 2018) package. Mantel test (vegan package, Oksanen et al., 2018) was applied to confirm whether differences between distant matrices of physicochemical properties and microbial communities of substrates are statistically significant (number of permutations = 999). To investigate the spread of data and detect clusters of OTUs, non-metric multidimensional scaling (NMDS) based on Bray–Curtis dissimilarity and fitted into two dimensions (*k* = 2) was done in PAST as well as R using the vegan package. To assure an appropriate representation of the data, only plots with a stress level < 0.2 were considered for further interpretation. The respective number of each replicate (1–3), the type of substrate, and the numeration of sample were included as explanatory variables. The core microbiome calculated by the get.coremicrobiome command in mothur was construed based on OTUs that are present in at least 80% of the samples. The sequences corresponding to the core OTUs were extracted from the dataset and manually assigned to the taxonomy using the standard nucleotide basic local alignment search tool (BLAST^®^, Altschul et al., 1990) and the search and classify function in SINA (Pruesse et al., 2012) using the latest SILVA database (v.132) and RDP II classifications (July 2017) (Cole et al., 2005). Figures were produced in R using the ggplot2 package (Wickham, 2016).

## Results

### Substrate Characteristics, Maturation, and Microbial Colonization

**FIGURE 1:**
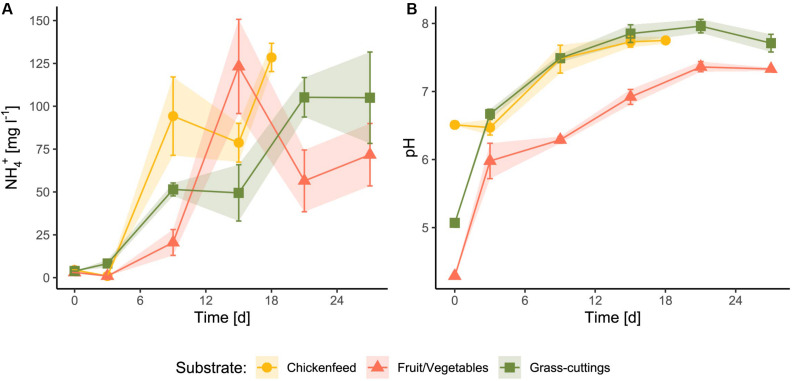
Changes in ammonium concentration **(A)** and pH levels **(B)** of the substrate after administration as feed for larvae (*n* = 3). Due to the larval activity, the substrate was mixed with indigestible residues, larval feces, and shavings.

The pH as well as protein and fat contents were higher in CF compared with the other two substrates (Table 1). In addition, dry matter content was markedly greater in CF than in GC and FV. The concentration of nitrogen compounds including ammonium and total nitrogen was approximately 50% lower in FV. After the substrates were administered to the feeding experiment, ammonium concentrations and pH therein were strongly affected by larval and microbial activity but showed no significant diet-dependent variation (NH_4_^+^: *p* = 0.876, pH: *p* = 0.334; Figure 1). The initial pH values (Day_0_) of the substrates were different and ranged from 4.3 in FV to 6.51 in CF. Both ammonia concentration and pH increased during the experiment with the highest mean NH_4_^+^ at 128 mg l^–1^ in CF and highest pH of approximately 8 in GC. Ammonium concentrations peaked in FV treatments on Day_15_, while they temporarily decreased in the other two treatments. The equations from linear trend-lines fitted to the curves delineated a stronger ammonium increase in CF treatments (*k* = 6.763) compared with the other two treatments (GC: *k* = 4.079, FV: *k* = 3.121), while the pH developed in a similar way (Supplementary Figure S1).

**TABLE 1:** Substrate characteristics, mass balance, degradation parameters, and mean compositional indicators of larvae and pupae (*n* = 3).

		Diet					

		Chickenfeed		Grass-cuttings		Fruit/vegetables	
		
		Mean	SD	Mean	SD	Mean	SD
Substrate characterization	pH	6.1	0.02	4.8	0.02	4.0	0.02
	Dry matter [%]	90.1	0.2	21.1	0.8	9.4	0.1
	Chemical oxygen demand [g l^–1^]	18.0	0.5	4.2	0.4	23.1	0.2
	Ammonium [mg l^–1^]	9.2	1.4	11.2	1.7	5.1	0.3
	Total carbon [%]	41.0	0.7	39.8	0.6	46.5	0.1
	Total nitrogen [%]	3.0	0.1	3.0	0.1	1.2	0.0
	Protein [mg ml^–1^]	3.6	0.2	0.6	0.1	2.0	0.1
	Fat [%]	3.6	0.2	0.7	0.1	2.9	0.2
Mass balance	Residues (Feed and feces)	38.6%	2.7%	50.2%	3.9%	40.0%	5.0%
	Larval and pupal biomass	28.0%	1.6%	9.6%	0.2%	12.2%	0.9%
	Larval metabolism	33.4%	2.5%	40.2%	3.9%	47.8%	5.0%
Degradation parameters	Waste reduction index (WRI)	1.7	0.1	1.2	0.1	1.5	0.1
	Efficiency of conversion of digested feed (ECD)	49.8%	2.0%	20.5%	1.8%	21.5%	1.8%
Larvae	Mean organic dry matter content	84.8%	1.4%	75.9%	0.5%	91.8%	1.8%
	Mean water content	67.9%	0.7%	71.4%	1.8%	66.0%	0.3%
Pupae	Pupation rate	94.1%	2.2%	70.7%	5.6%	83.8%	5.5%
	Mean organic dry matter content	83.8%	0.2%	76.2%	2.3%	88.7%	1.2%
	Mean water content	65.6%	0.6%	69.2%	2.3%	65.2%	1.1%

*The pupation rate was calculated based on total initial larvae pro box less the larvae removed for sampling. SD = standard deviation.*

**FIGURE 2:**
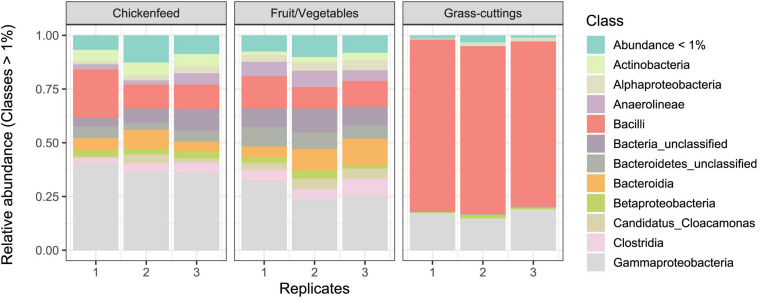
Relative abundance of bacterial classes in diets used as treatments (*n* = 3). OTUs with a relative abundance lower than 1% were summarized. Note that the significance of differences between sample groups was tested at fine scale delinetation, i.e., the level of genus and 97% OTUs. The community composition on class level is shown for simplicity instead of a more detailed scale.

Despite different matrices, the microbial profiling of the three diets used in the feeding experiment resulted in comparable sequence numbers (CF: 8.6 ± 0.5 10^4^ reads, FV: 8.02 ± 0.90 10^4^ reads, and GC: 6.7 ± 1.1 10^4^ reads). Data from initial diet samples before amendment into boxes were only subsampled and not further filtered due to the already low sequence numbers and were only used to deduce information about the prevalent microbiota in absence of larvae. In the CF and FV diets, a similar group of bacterial classes including Gammaproteobacteria (Enterobacteriaceae, *Morganella* sp.), Bacteroidia (*Dysgonomonas* sp.), and Bacilli (*Enterococcus* sp., *Lactococcus* sp.) account for most of the found sequences. In GC however, Bacilli (*Lactococcus* sp., *Lactobacillus* sp., *Weissella* sp.) and Gammaproteobacteria (Enterobacteriaceae, *Pantoea* sp.) were most abundant (Figure 2). The physicochemical characteristics and the microbial profile of substrate samples were compared using hierarchical clustering and significant differences between the matrices were confirmed by Mantel test (*p* = 0.001) (Supplementary Figure S2). CF and GC were more similar in their physicochemical properties in contrast to FV, but on the microbial community level, CF and FV showed fewer dissimilarities compared with GC.

### Larval Development Based on Distinct Diets

**FIGURE 3:**
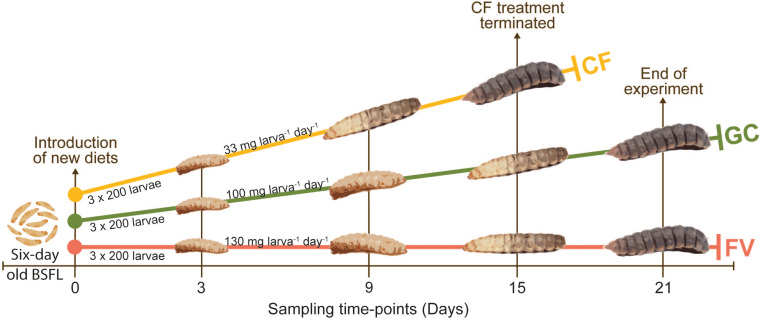
Illustration of the experimental design. Larvae were collectively raised on chickenfeed (CF) for 6 days. At Day_0_, 3 × 200 6-day old larvae were manually counted for each of the three diets and put into separate boxes. The two new diets (GC = grass-cuttings and FV = fruit/vegetable mix) were introduced to a triplicate of boxes each, while one triplicate was further fed with CF as control. The amount of feed per larva and day was adapted based on the respective organic and water content. CF treatment was terminated at Day_15_ as all larvae transitioned to prepupal stage. GC and FV treatment further continued until Day_21_.

The transitional phase before pupation was reached in the CF-treated replicates at Day_15_ and in the FV- and GC-treated replicates at Day_21_ (Figure 3). Larvae raised on CF outperformed the other two treatments, resulting in a higher biomass, more efficient degradation, and a higher pupation rate (Table 1). Larvae fed on CF showed a nearly three times higher gain in biomass compared with FV and GC, while the larvae raised on the two fresh substrates reduced and metabolized a higher proportion of substrate [input_total_ - (biomass_output_ + residues_total_) = metabolism_total_] but formed less biomass. Moreover, over the course of the feeding experiment, the efficiency of substrate degradation (WRI) was at least 15% higher, and the efficiency to convert substrate to larval biomass (ECD) was at least twice as high in CF fed larvae than in those fed with FV or GC. The amounts of residual matter at the end of the experiment were similar in CF and FV and were around 10% higher in GC. Water content in larvae and pupae was stable at approximately 67% across all treatments, while the organic matter was similar only between larval and pupal stage within each respective treatment. Larvae from the GC treatment exhibited a 8–10% lower content of organic dry matter in both developmental phases.

### Larval Gut Microbiome Dynamics and Identification of Core Community

The Illumina MiSeq read numbers of the homogenized larval gut samples ranged from 5.03 10^4^ to 1.63 10^5^ reads and were subsampled to the smallest sample size (4.5 10^4^ reads). Since removal of rare sequences from the bacterial data (OTUs with less than two sequences in all samples) did not suffice to denoise the subsampled dataset, a second step utilizing stronger filter removing all OTUs not present in at least five samples was applied.

**FIGURE 4:**
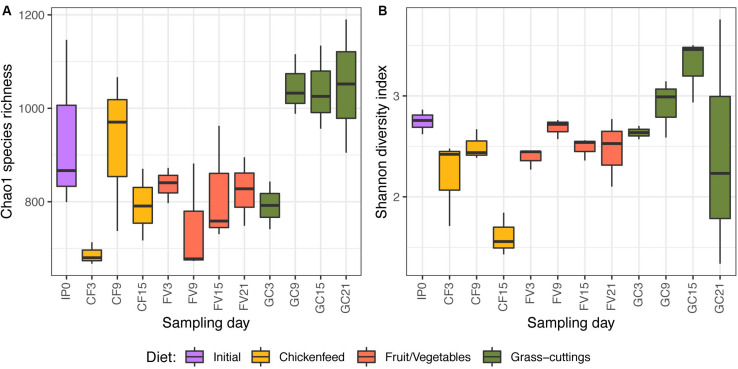
Chao1 species richness **(A)** and Shannon diversity index **(B)** of bacterial gut communities over time (*n* = 3). The lower and upper limitations of each box define the 25th and 75th percentile and the median is represented by the black lines. Labels on the *x*-axis describe the treatment (IP = initial population, CF = chickenfeed, FV = fruit/vegetable mix, GC = grass-cuttings) and the day of sample collection (0–21) (e.g., IP0 = gut samples from the initial population collected at t_0_).

Initial exploration of α-diversity (Figure 4) underlined a general impact of diet on the bacterial gut communities based on Chao1 species richness estimation (*p* = 0.002; Figure 4A) and Shannon diversity index (*p* = 0.012; Figure 4B). Both species richness and diversity decreased in CF-fed larvae between intermediate (Day_9_) and advanced (Day_15_) developmental stages. The same trend was found in community diversity of GC fed larvae, while species richness and diversity in FV treatments remained stable. Compositional deviations from the initial population (Day_0_) were not uniform throughout the treatments, and neither of the two indices pointed out significant temporal changes in the larval gut microbiomes.

**FIGURE 5:**
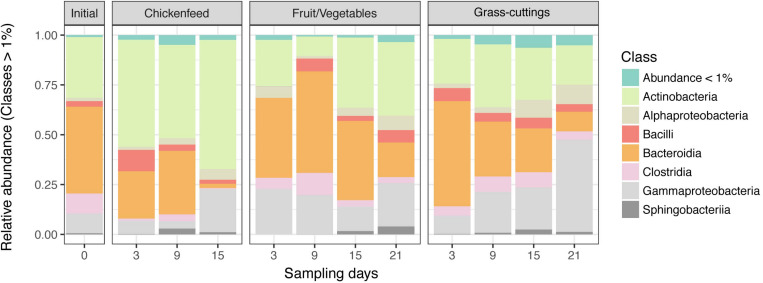
Relative abundance of bacterial classes in gut samples collected during the development of Black Soldier Fly larvae. Operational taxonomic units (OTUs) with a relative abundance lower than 1% were summarized. Note that the significance of differences between sample groups was tested at fine scale delinetation, i.e., the level of Genus and 97% OTUs. The community composition on class level is shown for simplicity instead of a more detailed scale. The microbiome composition of the initial population is derived from 6-day old larvae. All guts were analyzed in triplicates (*n* = 3).

A more detailed comparison of gut communities was carried out by analyzing the β-diversity. Triplicate gut samples taken at the respective time-points from the three treatments were considered as separate groups and statistically compared with each other using AMOVA, HOMOVA, and one-way NPMANOVA. To allow a confident description of relationships among variables, only statistical results confirmed by all three tests were considered valid. These pair-wise comparisons of groups resulted in no meaningful patterns in terms of statistically significant differences. Therefore, no substantial time- or diet-associated changes in the gut microbiota of the larvae can be deduced from the OTU data on this scale. Although the tests were able to detect single significant differences between some groups, they did not support biological deductions related to larval development and/or health. Predominant classes like Actinobacteria, Bacteroidia, and Gammaproteobacteria demonstrated time-dependent changes in their abundance. Especially in CF and GC, Gammaproteobacteria accumulated during larval development and reached a maximum at the last sampling time-point Day_21_, whereas Bacteroidia sequence numbers declined (Figure 5; for clarity, the community composition is presented on class level. A finer resolution on genus level can be found in Supplementary Figure S3). In the CF and FV treatment, changes in abundances of Bacteroidia and Gammaproteobacteria could be partly connected to the ingested substrates, since both diets contained up to 40% reads of Gammaproteobacteria and 12% reads of Bacteroidia.

**FIGURE 6:**
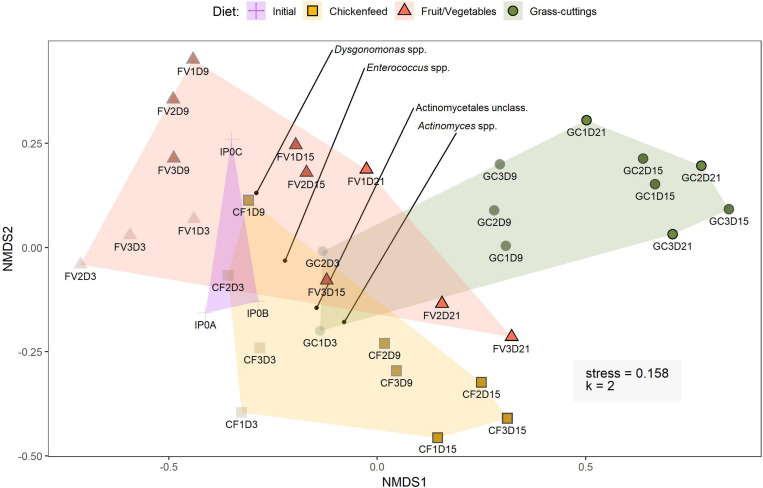
Bray–Curtis based non-metric multidimensional scaling of operational taxonomic units (OTUs) present in at least five samples. OTUs were clustered based on 97% similarity. The gradual increase in shading of data points indicates the progression from early to late sampling time-points (light = early, dark = late). Initial samples represent the initial larval gut microbiome composition at t_0_ before the introduction of new diets. The four black points indicate the distribution of core OTUs present in at least 80% of the samples. Sample labels consist of diet (IP = initial population, CF = chickenfeed, FV = fruit/vegetable mix, GC = grass-cuttings), the day of sample collection (0–21), and the replicate (1–3) (e.g., CF1D3 = chickenfeed fed larvae, first replicate collected at t_3_).

**FIGURE 7:**
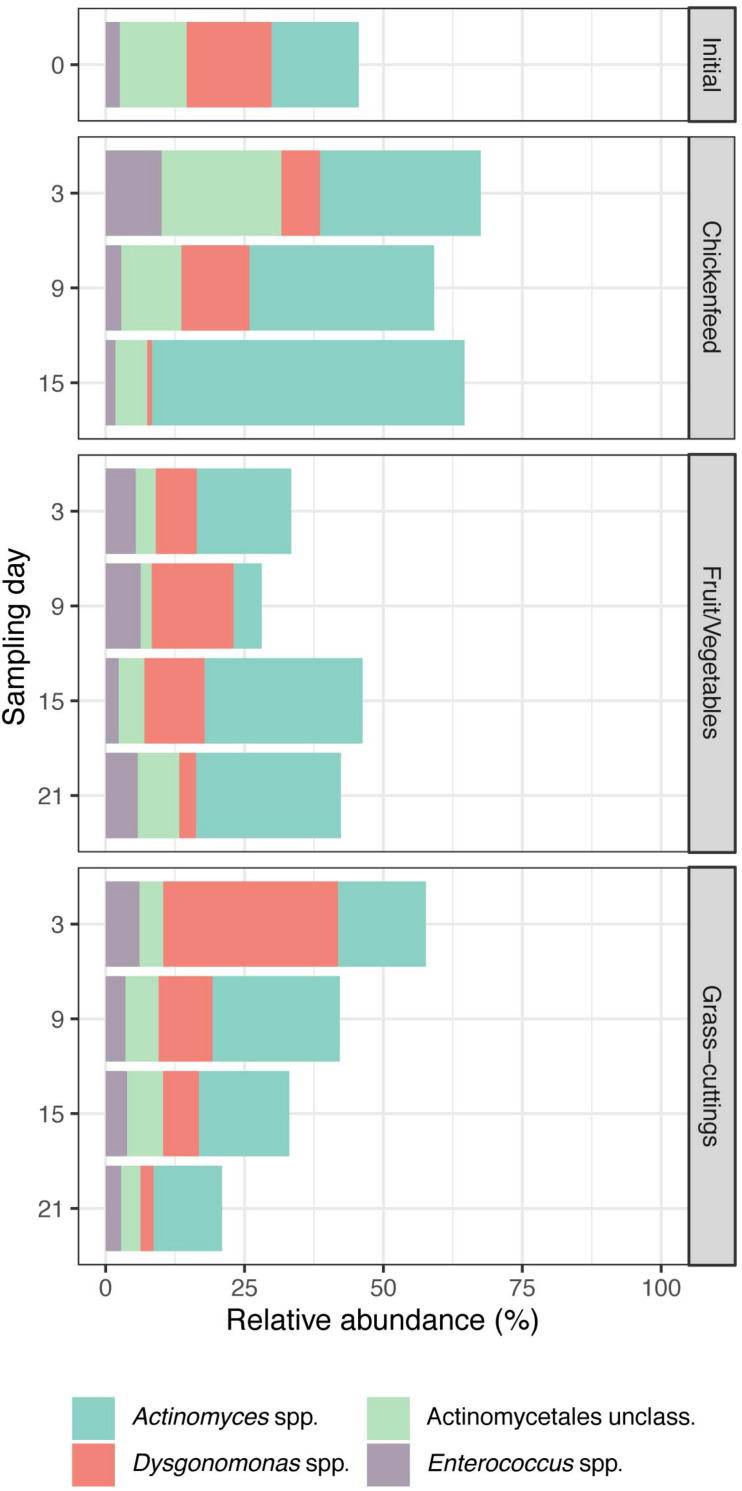
Relative abundances of OTUs identified as bacterial core community in the guts of Black Soldier Fly larvae and present in at least 80% of all samples (*n* = 3). Together, they account for approximately half of the reads generated during sequencing.

In a broader perspective, NMDS analysis (*k* = 2, stress = 0.158) on bacterial sequence data with sampling time-point and diet as grouping factors led to strong overlaps between the clustered treatment groups (Figure 6). The findings from these analyses are consistent in both *de novo* clustered OTU and reference-based phylotype datasets. Gut samples from 6-day old larvae (IP0A-C), representing the initial gut community before being subjected to new diets, are located near the center of the overlap and form a starting point for the divergent development of gut communities. Following an interpretation of the Pareto principle adapted to the context of microbial community dynamics (Dejonghe et al., 2001), bacterial OTUs present in at least 80% of the gut samples across the three treatments were considered members of the core community (Figure 7). Their location in the NMDS is indicated by black dots (Figure 6). These OTUs accounted for 44% of all sequences and were identified as *Actinomyces* sp., *Dysgonomonas* sp., and *Enterococcus* sp. and an unclassified representative of the order Actinomycetales. When lowering the threshold to OTUs present in at least 60% of the samples, the aforementioned list was extended by *Morganella* spp. and unclassified Enterobacteriaceae where this congregation accounted for 78% of all sequences. The results from the analysis were further confirmed by extracting the sequences corresponding to the OTUs and manually identifying them by BLAST^®^, SILVA, and RDP database search, which resulted in congruent results.

Archaeal sequences were present in very low numbers and made up approximately 0.02% of all sequences; therefore, neither subsampling nor filtering steps were applied prior to identifying the present archaeal representatives. They mainly were affiliated to the classes of Methanomicrobia (35%; *Methanosarcina* sp., *Methanoculleus* sp., *Methanotrix* sp., *Methanocorpusculum* sp., *Methanoregula* sp., *Methanospirillum* sp., *Methanosphaerula* sp., *Methanolinea* sp.), Methanobacteria (18%; *Methanobrevibacter* sp., *Methanobacterium* sp., *Methanosphaera* sp.), Thermoplasmata (16%; *Methanomassiliicoccus* sp., *Thermoplasmata* uncl.), and Nitrosopumilales (11%; *Nitrosopumilus* uncl.).

## Discussion

The aim of this study was to investigate the influence of three distinct diets (CF, GC, and FV mix) on the development of BSF larvae (BSFL) and to assess temporal changes in the composition of their microbial gut communities. The observations during this 21-day timeframe clearly showed that the composition of the diet strongly affected the thriving of BSFL in terms of developmental progress and biomass gain but did scarcely affect the composition of the gut microbiota. The use of 6-day old instead of freshly hatched larvae was a compromise between easier handling in counting and separation with the larvae still being in a susceptible stage of early development.

### Balanced Substrates Yield Higher Larval Biomass Gain

Due to its consistent commercial quality, CF represents a very balanced substrate ideal for providing a stable supply of a well-defined spectrum of nutrients and, therefore, for the maintenance of a population as well as for the use as control substrate. In previous studies, the benefits of using such diets to raise BSFL under laboratory conditions and to provide a reference substrate for feeding experiments have been described (Diener et al., 2009; Gold et al., 2018). Although all three diets used in our study were administered in the same intervals and contained an equalized amount of organic matter and water, larvae fed with FV and GC lagged behind in biomass gain, pupation rate, and degradation efficiency (Table 1). This indicates that a similar moisture and organic content were not sufficient to provide a foundation for developmental success. A well-adjusted content of macro- and micronutrients in the organic fraction of the diet is necessary for high degradation efficiency and biomass gain (Beniers and Graham, 2019). Although organic wastes and different types of manure are known for their heterogeneous composition, a guideline of optimal feeding rates, as defined by Diener et al. (2009) for standard fodder, should be advocated also for waste-derived substrates.

Diminished degradation, as also highlighted by a lower WRI, led to greater amounts of residues (up to 12% in GC) due to a higher content of recalcitrant components like cellulose especially found in grass (Rehman et al., 2017). This led to a reduced pupation rate (>90% in CF compared to 70% in GC) and a lower proportion of organics in both larvae and pupae when kept on a plant-based diet. On the contrary, larvae fed with FV exhibited the highest turnover of substrate. Compared with CF, FV diet ended up in similar amounts of residues suggesting an easy digestibility, although a lesser proportion was converted to biomass and was rather used for metabolism (Table 1). Depending on the amount of accumulating insect frass and residues (mixture of undigested substrates, excrements, and larval shavings), downstream treatment thereof in anaerobic digestion, pyrolysis for biochar production, or traditional composting could add value to insect rearing on a larger scale (Lalander et al., 2018; Yang et al., 2019). A similar sequential operation yielding an increased economic value from agricultural waste treatment by BSFL with subsequent composting of residues has been investigated by Zhu et al. (2012), while the direct use of the frass fraction as substitute to mineral fertilizer or vermicompost (Sarpong et al., 2019) has also been proposed as a viable option (Choi et al., 2009).

Both waste-related diets (GC and FV) could not keep up with the larval development and the biomass yield in CF. The high degradability and high protein content of CF rapidly affected the larval environment. Ammonia concentrations (Figure 1A) in CF-treated boxes increased fastest and were accompanied by a higher initial pH due to the innate properties of the raw feed (Figure 1B). Especially ammonium plays an important role during BSFL rearing, since it largely derives from organic nitrogen excreted by larvae and can further be transformed by associated gut microbes (Green and Popa, 2012). Partly due to this efficient microbial assimilation of nitrogen, emissions of nitrogen in form of greenhouse gases have been shown to be much lower in insect rearing compared with livestock breeding and traditional composting (Oonincx et al., 2010; Mertenat et al., 2019).

Based on hierarchical clustering, the characteristics of the diets varied, and they group differently at the physicochemical and microbial level. CF and GC were more similar to each other when compared by their physicochemical properties (Supplementary Figure S1A), while GC formed the outgroup on microbial community level (Supplementary Figure S1B). This is further illustrated in Figure 2, where especially Gammaproteobacteria made up for the major share of reads found in CF and FV but were outcompeted by Bacilli, mostly *Lactococcus* sp., *Lactobacillus* sp., and *Weissella* sp., in GC substrates.

Jiang et al. (2019) found that microbial communities in the added substrates were reshaped by gut bacteria excreted by larvae, leading to a similar microbiome in larval guts and their environment over time, while compositional changes within the gut were mainly induced by the ingestion of fresh substrates. In our trial, the composition of microbial communities was only determined in the fresh substrates to infer its direct relationship to the larval gut microbiome, and no monitoring of microbial substrate colonization in boxes during the experiment was carried out.

### Diet Drives Development of Larval Gut Microbiomes

Compared with direct effects on larval growth, diet effects were comparably small when looking at the larval gut microbiota. However, alpha diversity indicated a dietary impact on the gut microbiota both when based on Chao1 species richness estimation (Figure 4A) and Shannon diversity (Figure 4B). Not only was the microbial community in the GC diet considerably different from the other diets, but soon after its introduction as a diet it also favored the perpetuation of a comparatively higher species richness. Both GC and CF fed larvae exhibited reduced species diversity before pupation, possibly connected to a physical restructuring in the gut (Bruno et al., 2019).

The relative abundance of bacterial phyla described in this study were in line with previous similar studies (Jeon et al., 2011; Zheng et al., 2013) determining Actinobacteria, Bactoroidetes, Firmicutes, and especially Alpha- and Gammaproteobacteria within the phylum of Proteobacteria to be the major constituents in the BSFL gut (Figure 5). Comprehensive time- or diet-dependent changes were not detected at the β-diversity level. Sparse statistically significant dissimilarities in community compositions in larval guts from different time-points and treatments that were supported by all applied statistical tests (AMOVA, HOMOVA, one-way PERMANOVA) did not yield any meaningfully directed biological explanatory power. NMDS was used to spatially map samples in two dimensions and although previous statistical confirmation of dietary and temporal dynamics were weak, gut samples from more advanced developmental stages strayed further away from the state of initial gut microbiomes (Figure 6). However, bacteria already present in the gut seemed to compensate for the low microbial abundance in the fresh substrates used in this study. Thereby, the formative impact on the larval gut microbiomes was limited compared with more extensively colonized substrates often used in similar feeding experiments, such as organic wastes, animal manure, or human feces (Bruno et al., 2018; Cai et al., 2018).

The resilience of the resident gut microbiome in larvae from the three treatments could be connected to the age of the larvae when they were first exposed to the new diets. Using 16S rRNA pyrosequencing, Jeon et al. (2011) were able to measure distinguishing effects of various diets on the respective microbial communities in the gut of larvae that were exposed to different feeds directly after hatching. To increase the number of hatched eggs and to standardize the condition of hatched animals, the two new diets used as treatments were introduced into the experiments on the sixth day of the larval development, while a control group was further fed with CF. In this study, the 6 days of CF prior to exposure to new diets seem enough to allow for the establishment of resilient microbial communities through a “priming effect,” that is, the establishment of an indigenous population of gut bacteria that is not readily susceptible to colonization by allochthonous microorganisms taken up with the two newly introduced diets (Rillig et al., 2015), despite their measurable and different microbial fingerprint.

Due to the limitations of 16S rRNA amplicon sequencing (Poretsky et al., 2014), only analysis down to the genus level was performed, which could have left differences between treatments on lower taxonomic levels undetected. Other, higher resolution methods, such as whole genome shot-gun sequencing or metabolomic investigations could uncover dietary effects at strain and even functional gene level (Xia et al., 2017; Chen et al., 2018). Moreover, the gut microbiome of wild BSFL has not been studied yet, thereby limiting the possibility of comparing laboratory populations with fly populations accustomed to a changing diet over numerous generations. Larvae deriving from a lab population, and therefore, adapted to a consistent diet, could be less perceptive for the enrichment of transient microorganisms that found their way into their gut, where an observation of dietary effects over a timespan of multiple generations of flies could be necessary to illustrate a more comprehensive insight in gut microbiome dynamics. Microbial gut colonizers are crucial for the thriving of many insects suggesting that mechanisms have to be in place that avoid colonization by pathogens and favor the preservation of vital functional properties (Bahrndorff et al., 2016).

### A Microbial Core Community Provides Metabolic Foundation in the Gut

De Smet et al. (2018) and Wynants et al. (2018) recently proposed that, based on several bacterial OTUs found across larval guts exposed to various waste treatments, BSFL establish a core bacterial microbiome. However, they were still susceptible to microbial colonization coming from their surroundings, which was further described as “house flora.” The extensive repertoire of pathogen defense mechanisms inherent to BSFL allows the thriving of larvae also in hazardous habitats and is most likely in close relationship with gut-residing microbes (Vogel et al., 2018). To guarantee the dietary flexibility and availability of antimicrobial peptides in different environments, a stable group of bacteria able to provide such services needs to be present within the larvae. From our largely diet-independent community, we identified a core community that was present in at least 80% of the gut samples. Not only very few OTUs fulfilled this requirement but they also accounted for approximately half of all analyzed sequences (Figure 7). *Actinomyces* sp., *Dysgonomonas* sp., *Enterococcus* sp., and another unclassified representative of the order of Actinomycetales made up the core community and are well known for the role they play in (insect) guts (Dillon and Dillon, 2004; Engel and Moran, 2013; Dietrich et al., 2014).

The filamentous, high GC, Gram-positive, and mostly facultative anaerobic bacterium *Actinomyces* sp. can degrade a broad spectrum of organic material including lignin and chitin (Wang et al., 2014). It is often found as commensal in the gut of various animals, and the production of a variety of antibiotics inhibiting the growth of other microorganisms additionally represents a benefit for the larvae (Franke-Whittle et al., 2009; Hanning and Diaz-Sanchez, 2015).

*Dysgonomonas* sp. has mostly been known for its key role in the gut of termites during the degradation of recalcitrant lignocellulose but was also found in high abundances in the gut of BSFL (Yang et al., 2014; Sun et al., 2015). Bruno et al. (2019) highlighted the significance of *Dysgonomonas* in the digestion of complex polysaccharides, which was further emphasized by Jiang et al. (2019) on a functional level, indicating that *Dysgonomonas* sp. in the gut of BSFL is positively correlated with genes for sulfate, carbohydrate, and nitrogen metabolism. Shelomi et al. (2020) recently assessed the effect of post-production and post-consumer wastes on larval gut microbiota and pointed out multiple OTUs assigned to *Dysgonomonas* sp. as representatives of a core community, since they were found in the larval guts irrespective of the waste type. From a metagenomic analysis of the BSFL gut, the origin of a new α-galactosidase gene that makes it possible to break up α-galactoses abundant in non-digestible plant carbohydrates was traced back to a specific *Dysgonomonas* strain (Lee et al., 2018). This anaerobic bacterium is also able to contribute to the biodegradation of pharmaceutical products like ciprofloxacin when appearing in a consortium with other bacteria like, for instance, *Actinomyces* sp., underlining the potential for yet to discover biotechnological applications (Martins et al., 2018).

As a typical commensal gut colonizer, Gram-positive and facultative anaerobe *Enterococcus* sp. is involved in making nutrients accessible for the host and contributing to gut health (Dubin and Pamer, 2017). In other insects like the greater wax moth (*Galleria mellonella*), *Enterococus* dominates the microbial communities in the larval gut and supports their host by providing immunity-related antimicrobial peptides (Krams et al., 2017).

Considering the early stabilization of this core consortium of highly abundant microorganisms and the broad functional competences coming from its representatives, this specific gut microbiome in BSFL can be of great advantage when exposed to various nutrient-poor or even contaminated environments and for stable industrial production. Wynants et al. (2018) compared the BSFL gut microbiota from large-scale cycles and found that bacterial community composition and abundances generally differ among facilities but may in addition also vary between batches produced in those locations. Despite these variations linked to site-specific features including diverse diets, they identified a small group of bacterial genera shared across multiple rearing facilities. Besides *Pseudomonas* sp. and *Providencia* sp., this group also included *Enterococcus* sp. and *Morganella* sp., which our study pointed out as core members (found in 80 and 60% of the samples, respectively). The presence of these two genera further supports our approach to identify core members of the larvae’s gut microbiota.

Apart from that, the exposure to substrates that knowingly carry a high microbial bioburden such as organic animal wastes might represent a stronger driving force in shaping the larval gut microbiota than the low bioburden diets used in this study. In a comprehensive investigation, Zhan et al. (2019) identified 16 bacterial phyla of OTUs present in BSFL guts irrespective of having been fed food waste, poultry, dairy, or swine manure. The high abundances of Bacteroidetes and Proteobacteria in larval guts across diets and time coincide with our findings, since *Dysgonomonas* sp., *Actinomyces* sp., and the unclassified Actinomycetales identified in our analysis as members of the gut core community also belong to these two phyla. The absence of Firmicutes in our core community, however, may relate to the predominant function they take over in the degradation of animal manure. It is plausible that this phylum is not *a priori* inherent to BSFL but instead gets acquired when the larvae are exposed to manure.

If these observations are seen from a practical perspective, BSFL still demonstrated a great ability in degrading a broad variety of organic compounds, including various waste substrates, with the only two outputs being larval biomass and organic fertilizer in form of substrate residues and frass (Cickova et al., 2015). So far, no indication of the presence of a fungal core community has been brought forward since this kingdom has been largely neglected in microbiome studies. Observations of Boccazzi et al. (2017), however, suggest that diet is the sole driver of the BSFL gut mycobiome. Experiments sounding out the optimum amount of organic waste combined with commercially available well-balanced feeds such as CF provide room for improving the biological parameters relevant for large scale rearing (e.g., biomass gain, WRI, ECD, developmental time) and waste treatment. Even if the composition of wastes is inconsistent, a steadily available fraction of uniform quality CF in the diet could stabilize the degradation processes and boost the functional performance of the microbiome and the larva itself through the priming of the gut environment.

## Conclusion

This investigation sets an additional cornerstone in characterizing the gut’s microbiome and core community of *H. illucens* larvae, which are known for their ability to degrade a vast array of organic substances. Especially the industrial use of BSFL for animal feed production or waste management could profit from better controllable growth characteristics and a fast adaptation to changing diet compositions to guarantee consistent biomass gain and substrate degradation. Constant degradation rates under stable environmental conditions could allow adapting the number of larvae to the amount of substrate to be degraded, inhibiting the molding and/or dehydration of substrates. Fresh substrates like GC and FV with a low bioburden (compared with, e.g., matured biodegradable wastes) had no significant shaping influence on the microbial gut communities of 6-day old larvae, whereas BSFL rather maintain a stable gut microbiome already early in their development. This community is dominated by a simple group of highly abundant bacterial species (*Actinomyces* sp., *Dysgonomonas* sp., *Enterococcus* sp., unclassified Actinomycetales) known to play key roles in the degradation of organic substances.

## Data Availability Statement

The datasets generated for this study can be found in the European Nucleotide Archive (PRJEB33904).

## Author Contributions

AW and TB conceived the study design. AW performed the experiments together with CH, who also maintained the BSF population. FS, BS-S, and WA assisted in BSF maintenance. TK conducted the statistical and bioinformatical analyses and wrote the manuscript. BS, FS, BS-S, WA, and HI contributed scientific comments to the experimental design and the manuscript.

## Conflict of Interest

The authors declare that the research was conducted in the absence of any commercial or financial relationships that could be construed as a potential conflict of interest.
