# Supplementary figures and images for: The Core Gut Microbiome of Black Soldier Fly (Hermetia illucens) Larvae Raised on Low-Bioburden Diets
Source: Front Microbiol. 2020 May 21;11:993. doi: 10.3389/fmicb.2020.00993 (PMC7253588; doi:10.3389/fmicb.2020.00993)

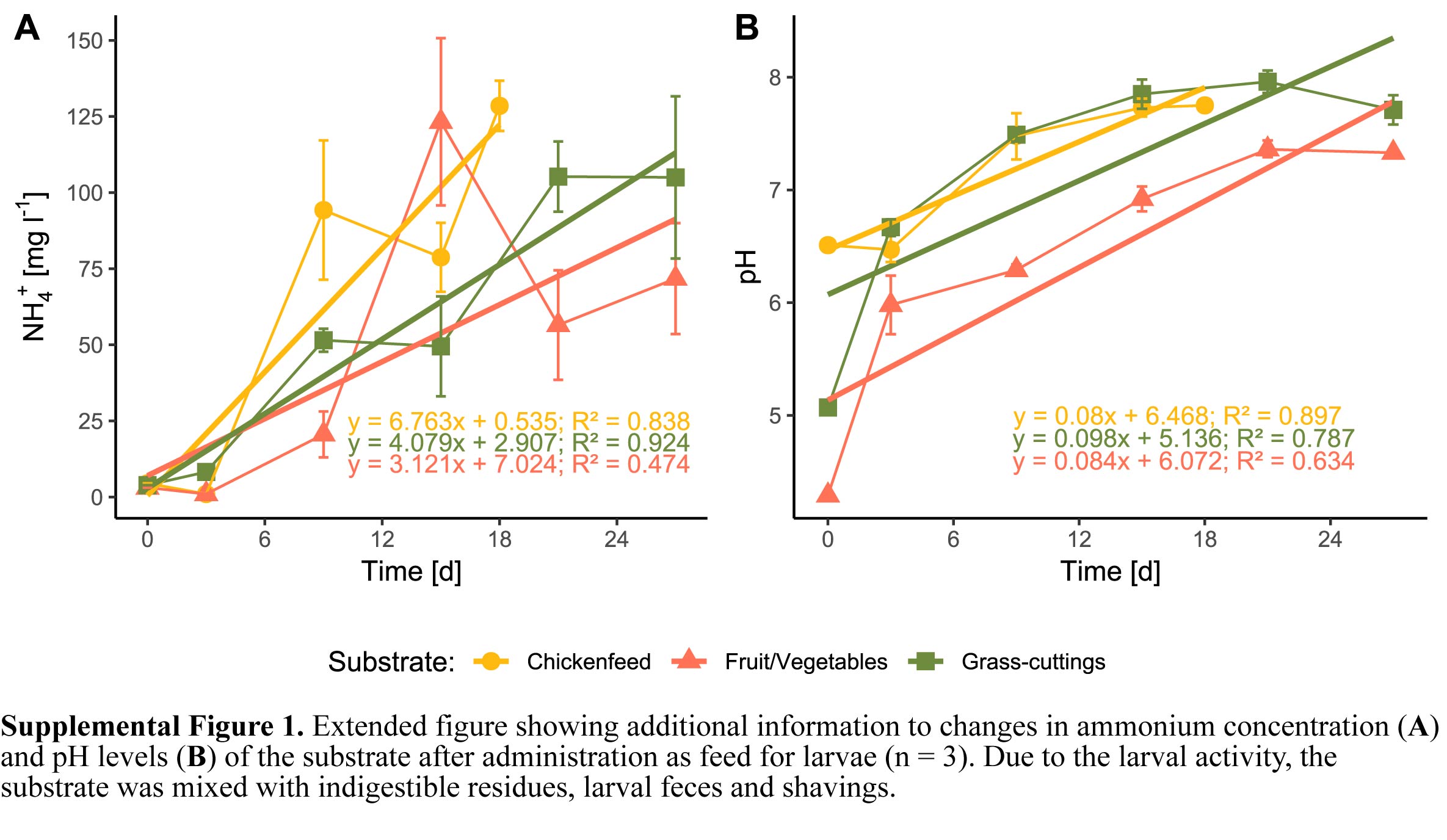

Supplement: Supplementary file 1 [file Image_1.JPEG]

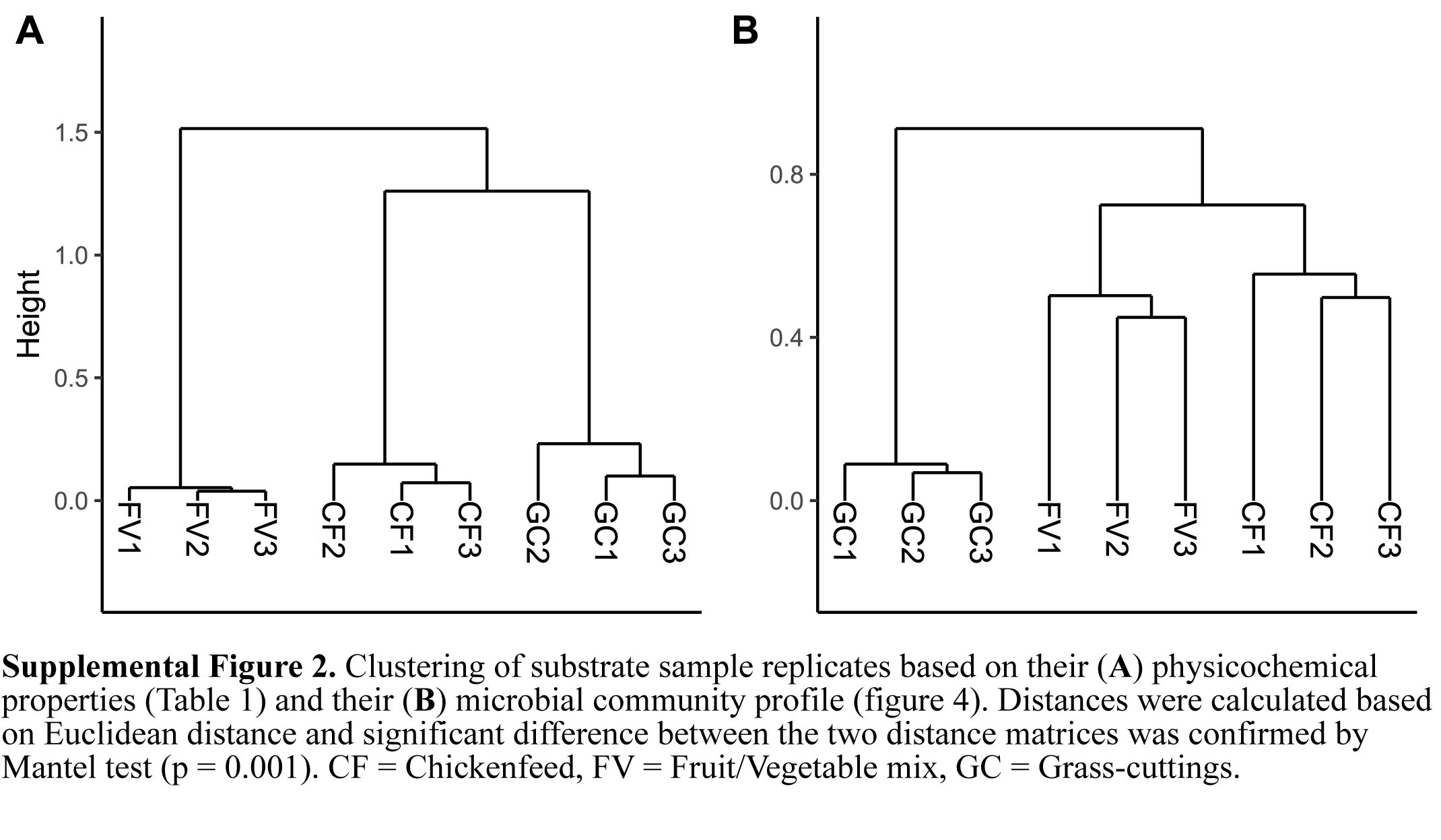

Supplement: Supplementary file 2 [file Image_2.JPEG]

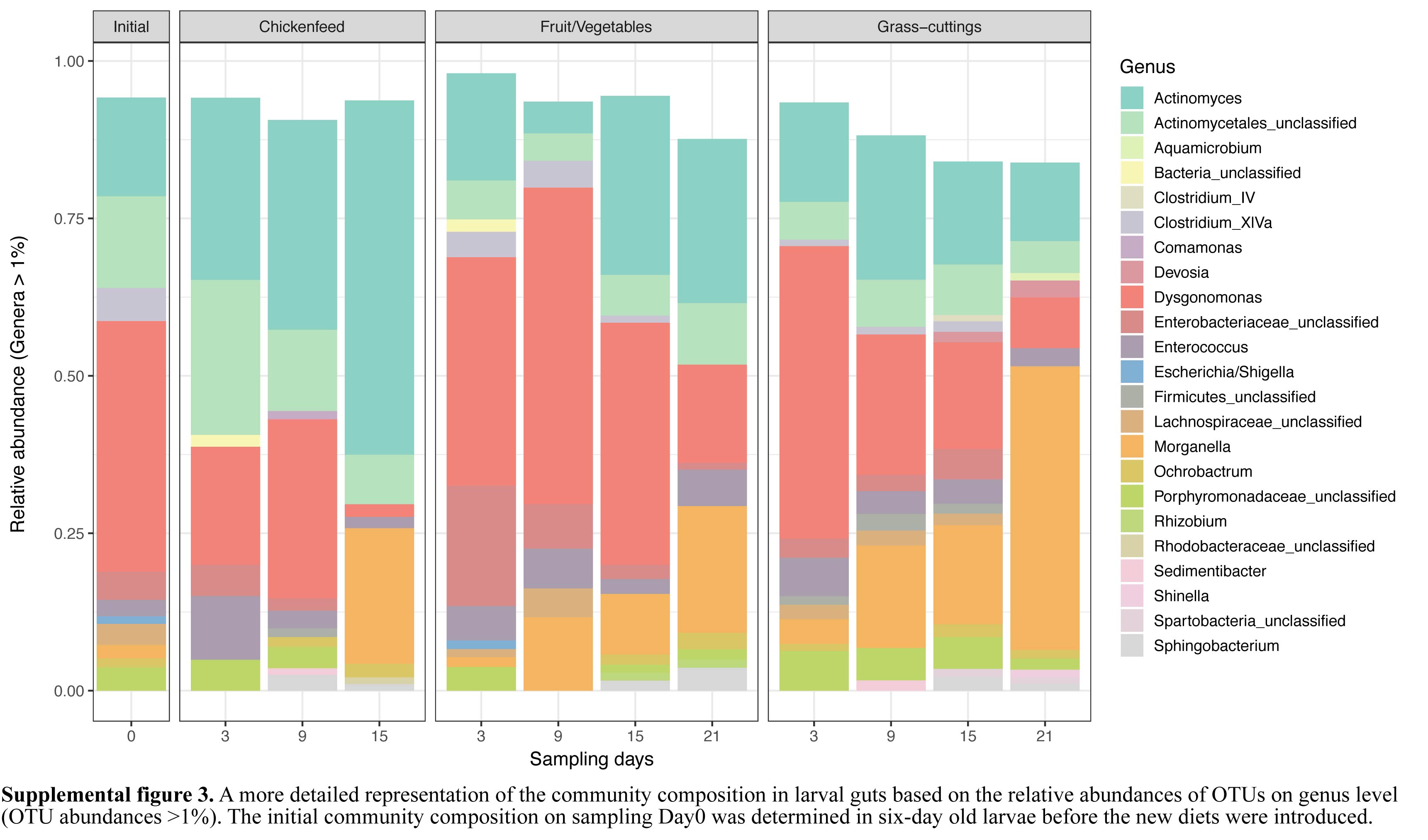

Supplement: Supplementary file 3 [file Image_3.JPEG]
